# A DNA vaccine candidate provides protection against Rift Valley Fever virus in sheep under natural field conditions

**DOI:** 10.3389/fcimb.2025.1628877

**Published:** 2025-08-26

**Authors:** Moufid Mhamadi, George (Giorgi) Babuadze, Aminata Badji, Marie-Edith Nepveu-Traversy, El Hadji Ndiaye, Alioune Gaye, Mignane Ndiaye, Moundhir Mhamadi, Frank William Mendy, Cheikh Talibouya Touré, Idrissa Dieng, Moussa Dia, Ndeye Sakha Bob, Marc-Antoine de La Vega, Ousmane Faye, Amadou Alpha Sall, Mawlouth Diallo, Gary Kobinger, Oumar Faye, Hugues Fausther-Bovendo

**Affiliations:** ^1^ Virology Department, The Pasteur Institute of Dakar, Dakar, Senegal; ^2^ Vaccine Research Center, Department of Preclinical Trials, The Institut Pasteur of Dakar, Dakar, Senegal; ^3^ Department of Microbiology and Immunology, University of Texas Medical Branch, Galveston, TX, United States; ^4^ Medical Zoology Department, The Pasteur Institute of Dakar, Dakar, Senegal; ^5^ Global Urgent and Advanced Research and Development, Batiscan, Quebec, QC, Canada; ^6^ DIATROPIX, The Pasteur Institute of Dakar, Dakar, Senegal; ^7^ Galveston National Laboratory, University of Texas Medical Branch, Galveston, TX, United States; ^8^ The Sealy Institute of Drug Discovery, University of Texas Medical Branch, Galveston, TX, United States

**Keywords:** RVFV, DNA vaccine, electroporation (EP), sheep, field study, neutralizing antibodies (NAbs), glycoprotein precursor, veterinary vaccine

## Abstract

Rift Valley Fever virus (RVFV) is a mosquito-borne zoonotic pathogen, that causes significant morbidity and mortality in livestock, including high abortion rates in pregnant animals and elevated case fatality in neonates, representing a major threat to both animal and human health. Vaccination is the most effective countermeasure to reduce RVFV’s impact. In this study, we designed a veterinary DNA vaccine encoding a consensus RVFV glycoprotein precursor (GPC), optimized for expression in sheep. The construct was evaluated for immunogenicity in mice and sheep and for protective efficacy in sheep raised under natural field conditions in Senegal, West Africa. The vaccine induced robust humoral responses characterized by high neutralizing antibody titers in both mice and sheep. Under natural exposure, vaccinated sheep showed reduced infection rates (3.2%) compared with controls (14.3%), and neutralizing antibody responses persisted for more than one year. Importantly, the vaccine was well tolerated, including in pregnant animals, with no adverse outcomes such as abortions or fetal abnormalities. These findings demonstrate that a DNA-based RVFV vaccine can elicit durable immunity and provide protection in livestock under real-world conditions. This study highlights the potential of DNA vaccines as a safe, effective, and affordable alternative to existing veterinary vaccines and supports their further development as a key strategy to reduce RVFV transmission and improve animal and human health outcomes in endemic regions.

## Introduction

Rift Valley Fever virus (RVFV) is a mosquito-borne zoonotic virus. It is included on the World Health Organization`s (WHO) list of priority diseases due to its large outbreak potential (Nair et al., 2023; Organization, W.H, 2024). While RVFV is endemic in multiple countries across Africa and the Arabian Peninsula, the geographic distribution of competent vectors, including *Aedes* and *Culex* mosquitoes, extends beyond these regions and is steadily increasing due to rising global temperatures (Tinto et al., 2023). In humans, RVFV infection mainly causes self-limiting febrile illnesses but can be severe, and even fatal, particularly in neonates (Ikegami and Makino, 2011; Nair et al., 2023). RVFV outbreaks have devastating economic consequences. Infection in pregnant livestock leads to abortions, stillbirths and/or fetal deformities while infection in young animals is particularly lethal, with reported case fatality rates exceeding 90% for neonates (Nair et al., 2023; Tinto et al., 2023). Beyond the loss of animal lives, subsequent bans on livestock exports can cost affected communities tens of millions of US dollars (Nair et al., 2023; Tinto et al., 2023). Preventing RVFV outbreaks could not only improve human and livestock health, but enhance food security and the economic stability of affected areas (Kimani et al., 2016).

**Figure 1:**
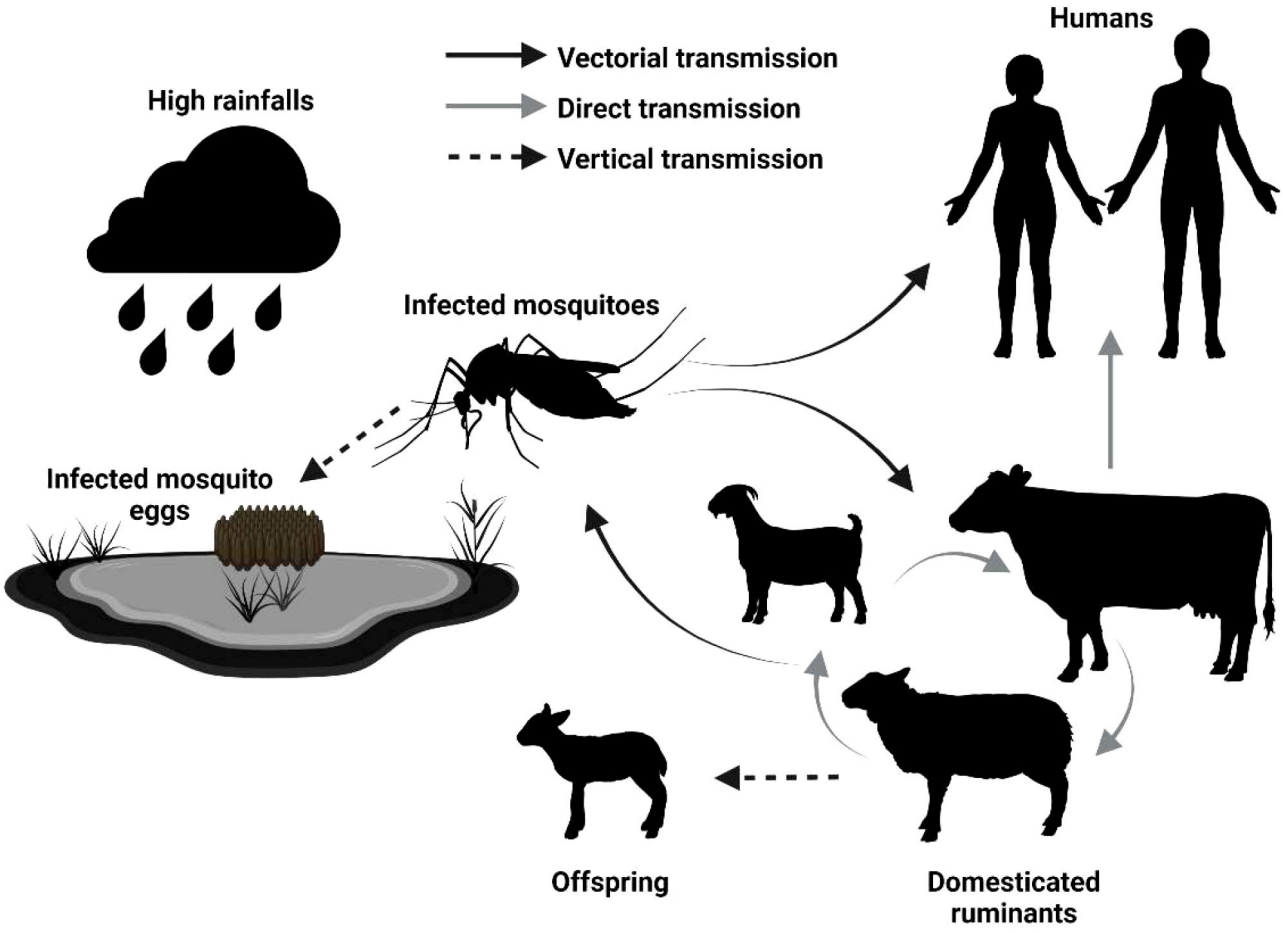
Epidemiological cycle of Rift Valley Fever Virus. High rainfall and flooding trigger the hatching of mosquito eggs, including those infected with RVFV, leading to an increase in infected mosquito populations. These mosquitoes feed on livestock (domesticated ruminants), amplifying the virus and raising the risk of direct transmission to other animals, including humans. The types of transmission are indicated by the full black arrow (vectorial transmission), full gray arrow (direct transmission), and dotted black arrow (vertical transmission, from parent to offspring), respectively [modified from (Jourdain et al., 2019)].

The epidemiological cycle of RVFV primarily involves domestic ruminants (e.g., sheep, cattle, goats) and zoophilic, floodwater-breeding *Aedes* mosquitoes, with *Culex* mosquitoes acting as secondary vectors. As depicted in **Figure 1**, humans can become infected either through mosquito bites (vectorial transmission) or direct contact with infected animals, particularly during slaughtering or birthing activities, the latter being the most common route of infection (Nair et al., 2023; Nicholas et al., 2014). Currently, no approved human vaccine against RVFV exists. However, the use of veterinary vaccines to reduce RVFV infections in animals holds the potential to significantly lower the risk of spillover to humans by controlling the spread of the virus in livestock populations (Alkan et al., 2023; Nair et al., 2023). Veterinary vaccines based on live attenuated or inactivated virus are currently licensed in several African countries. Formalin-inactivated RVFV vaccines were produced and sold, but their limited immunogenicity and the need for booster doses have hindered their widespread adoption (Alhaj, 2016). Numerous generations of live attenuated vaccines have been developed for veterinary use against RVFV (Alkan et al., 2023; Sindato et al., 2021). The new generation of live attenuated vaccines remains effective after a single dose and is no longer associated with abortion and fetal deformities. However, the risk of reversion to virulence remains a concern (Bird et al., 2008; Ikegami and Makino, 2011; Wichgers Schreur et al., 2021). Novel RVFV vaccines based on diverse vaccine platforms, including viral vectors, subunits and DNA, have shown promising results in animal infection models (Kitandwe et al., 2022). Despite these laboratory successes, they have yet to lead to vaccine licensure for veterinary use (Alkan et al., 2023; Nair et al., 2023).

Here, we report the development of a safe, DNA-based veterinary vaccine candidate against RVFV. The immunogenicity in mice and sheep, as well as the protective efficacy of the vaccine in sheep raised under natural field conditions, were evaluated.

## Materials and methods

### RVFV DNA vaccines

A synthetic gene encoding a 1,068-amino acid consensus polyprotein of the Rift Valley Fever Virus (RVFV) glycoprotein precursor (GPC) was designed based on the full M segment open reading frame (ORF), starting from the fourth in-frame methionine (AUG). This design excludes the upstream NSm and LGp regions and preserves the native polyprotein architecture that is post-translationally cleaved into the mature Gn and Gc glycoproteins. Similar constructs have been shown to elicit protective immunity *in vivo*, including in DNA-vaccinated mice challenged with RVFV-MP12 (Heise et al., 2009; Lorenzo et al., 2010; Warimwe et al., 2013). To enhance secretion efficiency, a synthetic signal peptide (MAGIAMTVLPALAVFALAPVVFA) was fused to the N-terminus.

To maximize cross-strain immunogenicity, a majority-rule consensus sequence was generated by aligning all publicly available RVFV M segment sequences in the GenBank database as of April 2021. Over 150 full-length and partial sequences were included, representing a diverse range of geographic and temporal isolates. Representative strains used in the alignment included Smithburn (NCBI: DQ380193), ZH-548 (NCBI: DQ380206.1), MP-12 (NCBI: DQ380208.1), Clone 13 (NCBI: DQ380213.1), and Mauritania-2010 (NCBI: KM210509). The final construct spans the complete GPC, including both Gn and Gc ectodomains, their transmembrane regions, and cytoplasmic tails.

**Figure 2:**
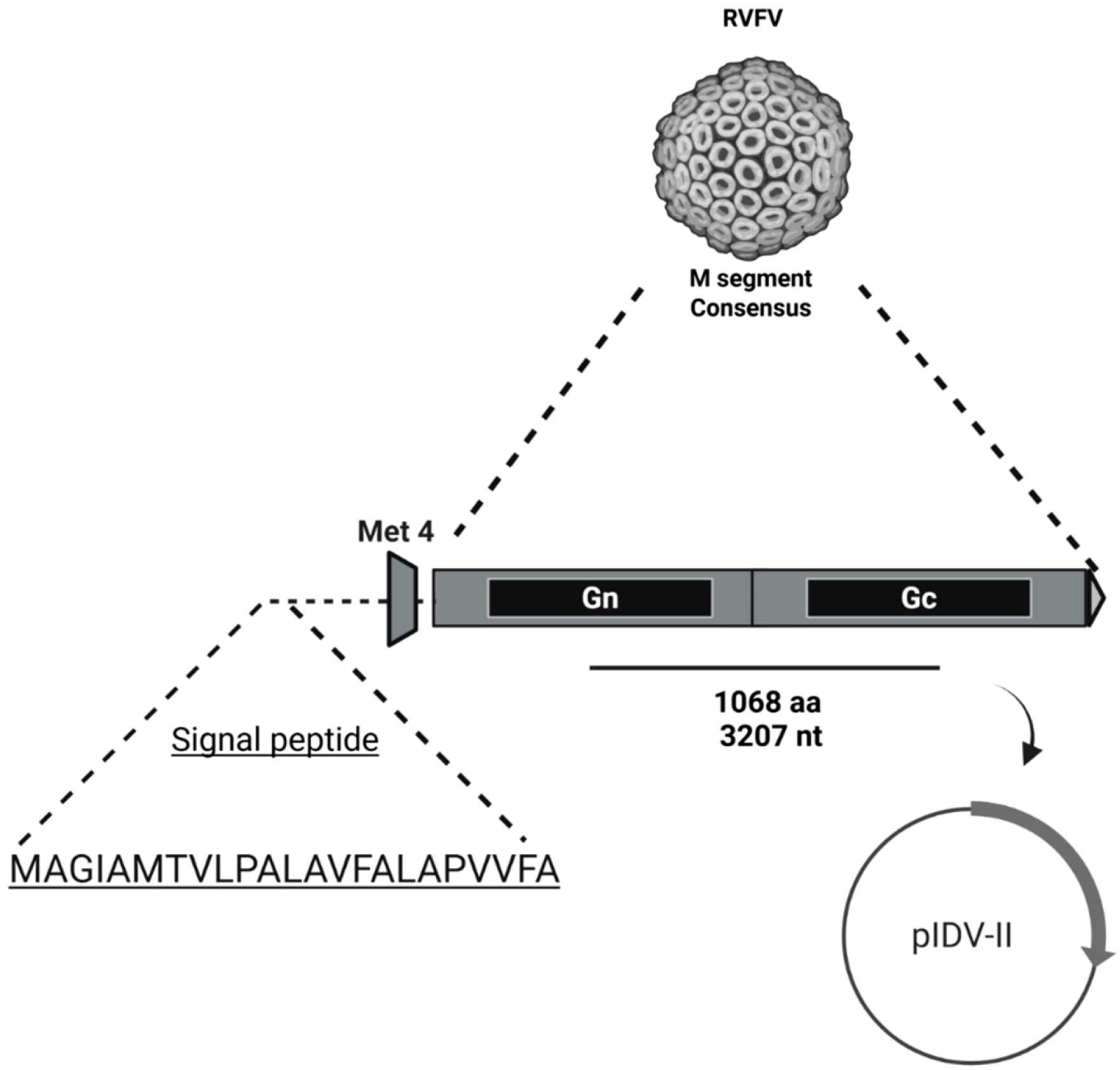
Schematic representation of the synthetic consensus RVFV M segment glycoprotein precursor (GPC) construct cloned into the pIDV-II DNA vaccine vector. The construct encodes a 1,068-amino acid consensus GPC sequence (3,207 nucleotides), derived from the M segment open reading frame starting at the fourth in-frame methionine (Met4), thereby excluding the upstream NSm and LGp regions. A synthetic signal peptide (MAGIAMTVLPALAVFALAPVVFA) was fused to the N-terminus to promote secretion of the translated polyprotein. The gene was codon-optimized for expression in sheep and inserted into the eukaryotic expression vector pIDV-II.

The finalized amino acid sequence was reverse-translated and codon-optimized for expression in sheep (*Ovis aries*). The gene was synthesized (GenScript, Piscataway, NJ) and cloned into the pIDV-II DNA vaccine vector under control of the CAG promoter, as previously described (Babuadze et al., 2021). A schematic of the construct is shown in **Figure 2**. The insert was fully verified by Sanger sequencing to confirm the absence of errors introduced during synthesis or cloning.

### Animal experiments

All mice experiments complied with the Canadian Council on Animal Care guidelines and were approved by the Animal Care Ethics Committee located at the Université Laval under research protocol number 2016096-1. Sheep experiments were approved by and conducted in accordance with the National Ethical Committee for Health Research in Senegal (# 00000806MSAS/DPRS/DR).

### ELISA

For serological analysis, sheep sera were inactivated for 1 hour at 56°C, while mice sera were use untreated. Ninety-six-well plates were coated overnight at 4°C with 50 ng per well of RVFV Gn (40338-V08B, Cedarlane Laboratories, Burlington, Canada) or nucleoprotein (ab318941, Abcam, Waltham, MA). After washing, the plates were blocked with 5% milk in Phosphate Buffer Saline (PBS) at room temperature for 2 hours. End titer dilutions were achieved for mice sera while 1:100 dilution was used for sheep sera, followed by an incubation of 1 hour at 37°C. Following extensive washes, the plates were incubated with horseradish peroxidase (HRP)-conjugated anti-mouse IgG or anti-bovine IgG, both from Mandel Scientific (Guelph, Canada). After additional washes, ABTS substrate (Mandel Scientific) was added, and absorbance was measured at 405 nm.

### RVFV RT-qPCR

RVFV screening by RT-qPCR was conducted as previously reported (Mhamadi et al., 2023). RNA from sheep blood samples was extracted using the QIAamp RNA Viral Kit (Qiagen, Heiden, Germany), following the manufacturer’s instructions. The quality and quantity of the extracted RNA were assessed with a NanoDrop spectrophotometer (Thermo Fisher Scientific), ensuring A260/A280 ratios between 1.8 and 2.0. RVFV genomic fragments were detected using the AgPath-ID One-step RT-PCR kit (Thermo Fisher, Burlington, Canada) with 2 μL of RNA template.

Previously reported primers (TGCCACGAGTYAGAGCCA and GTGGGTCCGAGAGTYTGC) and probe (TCCTTCTCCCAGTCAGCCCCAC) specific to the non-structural (NSs) gene were used (Mhamadi et al., 2023; Weidmann et al., 2008). The RT-qPCR cycling conditions were as follows: 10 min at 50°C, 15 min at 95°C, followed by 40 cycles of 15 sec at 95°C and 1 min at 60°C. Positive and negative controls were included in each run, and the presence of RVFV was determined by a Ct value below 35.

### RVF-GP pseudotyped lentiviruses neutralization assay

To generate RVF-GP-pseudotyped lentiviruses, 293T/17 cells (ATCC, CRL 11268) were co-transfected with 3 μg of pIDV-II-RVF-GP, 3 μg of psPAX2 (Addgene, Watertown, MA #12260), and 4 μg of pHAGE-CMV-Luc2-IRES-zsGreen (Addgene #164432) using polyethylenimine (PEI25K, Polysciences, Warrington, PA). Supernatants were harvested 72 hours post-transfection and filtered through a 0.45 μm filter. The specificity of the assay was evaluated prior to the assessment of samples using commercially available neutralizing antibodies (Monoclonal Anti-Rift Valley Fever Virus Gn Glycoprotein, Clone 4D4, BEI Resources NR-43190) and negative controls, such as RVFV-negative serum.

Mice serum samples were collected on days 40 and 80, while sheep serum used in the neutralization assay were collected on days 56, 146 and 356. Control samples were obtained from unvaccinated animals. For the assay, twofold serial dilutions of each serum sample were mixed with a constant volume of RVF-GP-pseudotyped lentiviruses. After a one-hour incubation, the pseudotyped virus-serum mixtures were transferred to HEK 293 cells that had been seeded (3 × 10^4^ cells/well) the previous day in white 96-well plates (Greiner Bio-One, Monroe, NC #655083). After 72 hours, luminescence was measured using Bright-Glo (Promega, Madison, WI, #E2650) and a microplate reader (Biotek Synergy). The reciprocal of the serum dilution required to reduce the number of infected cells by 90% relative to the virus-only control was recorded as the 90% neutralizing titer or inhibition dose (ID90).

### RVFV neutralization assay

To screen for previous RVFV infection in livestock, neutralizing antibodies were detected using an adapted version of a previously described and validated in-house plaque reduction neutralization test (PRNT), originally developed for yellow fever virus diagnostics and vaccine clinical trials (Dia et al., 2023)​. Briefly, inactivated sera (60°C for 20 min) were incubated for 1 hour with approximately 1000 plaque forming units (PFU) of RVFV (Smithburn Strain). All samples were screened at a single serum dilution (1:10). The sera/RVFV mixtures were added to monolayers of porcine stable (PS) kidney cells (6 × 10^4) in 96-well plates. After 4 hours of incubation at 37°C, the cell monolayers were washed and overlaid with L-15 media supplemented with 0.6% carboxymethyl-cellulose and 3% FBS. Four days later, the cells were washed and stained with Amido Black (Sigma-Aldrich)., which both stains and fixes living cells in a deep blue color. PFU resulting from viral replication and cell lysis appeared as distinct white, round spots (Dia et al., 2023). A representative image of the plaque reduction neutralization assay is shown in **Supplementary Figure 1**.

### Mice immunization

Five- to six–week-old female BALB/c mice (*n* = 6), purchased from Charles River (Laval, Canada), were immunized 40 days apart with 100 µg of plasmid DNA encoding the RVFV vaccine construct. Each immunization consisted of 100 µg of DNA in 100 µl (50 μl per limb) of endotoxin-free Tris-EDTA (TE) buffer, delivered intramuscularly into the caudal thigh muscle. Mice were bled via the saphenous route on days 40 and 80. Sera from naïve mice, collected prior to vaccination, were used as controls.

### Sheep immunization study

The study was conducted at our surveillance site in Agnam, located in northeastern Senegal, West Africa (Mhamadi et al., 2023). Local sheep were screened for previous RVFV infections by ELISA, live RVFV neutralization assays and RT-qPCR as described above. Sheep were considered RVFV naïve if they tested negative for IgG against the RVFV nucleoprotein by ELISA, showed no neutralization at a 1:10 dilution, and had undetectable RVFV RNA by qRT-PCR (Ct value above 35).RVFV-naive animals received two doses of 1 mg (2 mg total) of DNA vaccine or 1 mL of endotoxin-free TE buffer intramuscularly, followed by electroporation using the CELLECTRA^®^-3P device (Inovio, San Diego, CA). The device delivered three pulses of 0.2 A constant current, each lasting 52 milliseconds with 3 second intervals between pulses, using a 3-needle electrode array inserted approximately 3–5 mm into the muscle. Immunizations were administered 28 days apart. All animals were bled via the jugular route throughout the experiment. IgG antibody responses against RVFV Gn were measured to evaluate the vaccine-induced humoral response, while detection of IgG antibodies against RVFV Nucleoprotein (N) was used as a surrogate for natural infections. At the end of the study, all surviving animals were transferred to the Pasteur Institute of Dakar ‘s sheep flocks and are being kept alive in the IPD’s animal herds. These animals may be used in other programs or as a continuation of these vaccine studies.

### Statistical analysis

GraphPad Prism (version 10) was used for statistical analysis. The magnitude of the generated antibody response in mice was compared using an ordinary one-way ANOVA followed by Sidak’s multiple comparisons test. The neutralizing potency in immunized mice was analyzed using an ordinary one-way ANOVA followed by Dunnett’s multiple comparisons test, while an ordinary one-way ANOVA followed by Tukey’s multiple comparisons test was used for the sheep neutralizing assay analysis. Protection from RVFV infection was analyzed using a Mantel-Cox test.

## Results

### Vaccine development

**Figure 3:**
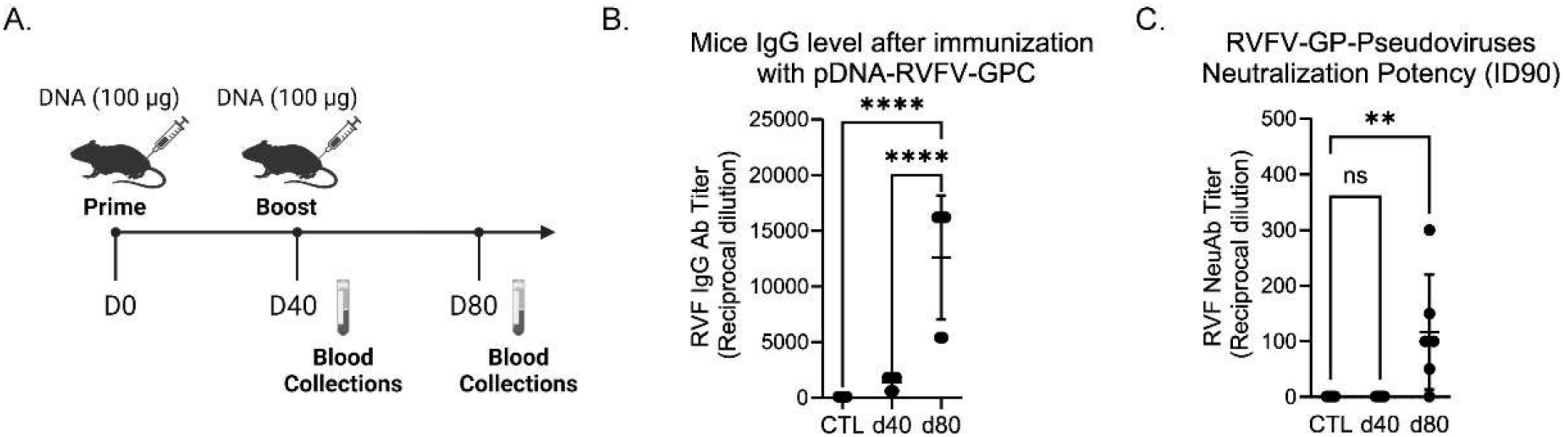
RVFV antibody response in immunized mice. Female BALB/c mice (5–6 weeks old, n = 6/group) were immunized intramuscularly with 100 µg of pDNA-RVFV-GPC plasmid DNA. **(A)** Immunization timeline showing vaccination and blood collection timepoints. **(B)** IgG antibody responses to Gn RVFV glycoprotein, measured by ELISA at days 40 and 80. Values represent reciprocal dilutions, with p-values < 0.0001 indicated by ****; ns indicates not significant. **(C)** Neutralizing potency measured using RVFV pseudotyped lentiviruses and a luciferase reporter system. The control group (CTL) sera from naïve mice and sera collected on days 40 and 80 after vaccination, were tested for their capacity to neutralize 90% of infection (ID90). Values represent reciprocal dilutions from two independent experiments (n = 2), with a p-value of 0.0078 indicated by **.

We hypothesized that DNA-based vaccines could serve as suitable veterinary vaccine candidates against RVFV. To test this hypothesis, we generated a consensus sequence of the RVFV glycoproteins (GPC), sheep codon-optimized it for increased expression in livestock, and cloned it into pIDV-II, a novel plasmid developed for DNA based immunization (Babuadze et al., 2021). The immunogenicity of this vaccine, designated pDNA-RVFV-GPC, was first evaluated in mice, which were immunized intramuscularly (IM) with 100µg of DNA per dose, administered 40 days apart (**Figure 3A**). The resulting humoral response was assessed by ELISA. After a single dose, RVFV (strain MP-12) Gn-specific IgG levels were low. However, following a boost with pDNA-RVFV-GPC on day 40, the antibody response increased significantly (p< 0.0001), with endpoint titers rising from 1,400 to 12,600 (**Figure 3B**). The sample size was calculated using Cochran’s formula: *n = t² × p × (1 - p)/m².* Where *t* = 1.96 (95% confidence), *p* = 2.3% prevalence (from unpublished local data), and *m* = 0.05. This yielded a required sample size of 35 animals per group.

The neutralizing capacity of the generated antibodies against RVF infection was evaluated using a pseudovirus neutralization assay. This assay uses pseudotyped-lentiviruses expressing RVFV glycoproteins on their surface, mimicking viral entry into target cells (in this case, HEK cells). Successful infection leads to luciferase expression, enabling the quantification of neutralization efficacy. The reciprocal titer dilution corresponding to 90% neutralization increased significantly after the boost, rising from 1 to 116.9 ± 42.05 SEM (**Figure 3C**).

### Vaccine efficacy study in field conditions

The immunogenicity and protective efficacy of the pDNA-RVFV-GPC vaccine were rigorously evaluated in a sheep model. Our previous research indicated a high prevalence of Rift Valley Fever virus (RVFV) among livestock in the Northeastern region of Senegal, West Africa (Mhamadi et al., 2023). Building on these findings, we conducted a comprehensive screening of sheep from this area using RVF nucleoprotein (N) ELISA, RT-qPCR and live RVFV PNRT to assess both current and past RVFV exposure. From this population, 70 RVFV-naïve sheep (22 males and 48 females), confirmed to have no N-specific antibodies, no neutralizing antibodies against RVFV and no signs of infection by RT-qPCR, were carefully selected and randomized into two groups (*n =* 35 animals/group). This sample size provides reasonable statistical power under field conditions, where a larger number of animals helps account for increased variability, including sex differences and heterogenous exposure risk. The experimental group received two doses of the pDNA-RVFV-GPC vaccine, administered intramuscularly 28 days apart, followed by electroporation to enhance immunogenicity (Kisakov et al., 2024). The control group received an equivalent volume of TE buffer. All sheep in this study were housed alongside other animals present at the surveillance site in Agnam, including goats, cattle, and horses. Notably, the study began on May 24, 2022, approximately six weeks before the start of the rainy season, which typically begins in mid-July. RVFV surveillance conducted in 2021 in the same area indicated that infections in both humans and livestock primarily occurred during the rainy season, between July and October, with a peak between September and October (Mhamadi et al., 2023).

**Figure 4:**
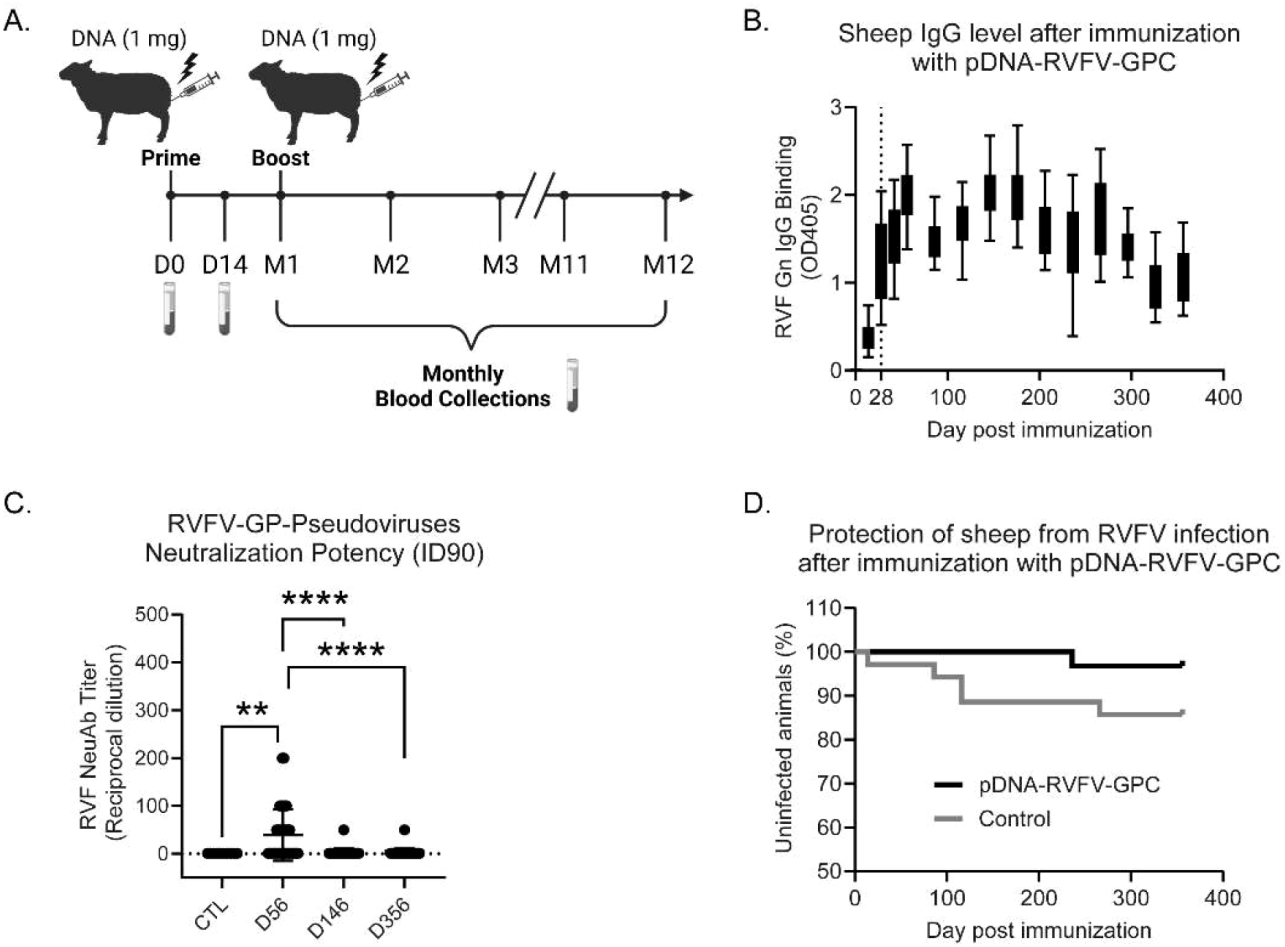
Immunogenicity and protective efficacy in vaccinated sheep. Local sheep from Senegal (West Africa) were immunized intramuscularly, followed by electroporation with either PBS (control, n = 35) or two 1 mg doses of pDNA-RVFV-GPC (n = 31). **(A)** Immunization and sampling timeline. **(B)** IgG antibody response specific to the RVFV Gn glycoprotein measured by ELISA. Data represent optical density at a 1:100 serum dilution over one year. **(C)** Neutralizing potency measured using RVFV pseudotyped lentiviruses and a luciferase reporter system. Control group (CTL) sera from naïve animals and sera collected on days 56, 146 and 356 post-vaccination were tested for their capacity to neutralize 90% of infection (ID90). Values represent reciprocal dilutions. P-values < 0.0001 are indicated by ****; a p-value of 0.0061 is indicated by **. **(D)** Percentage of uninfected sheep over time, showing the protection conferred by vaccination throughout the experiment.

Both groups were kept under natural field conditions and bled before and after the first injection, then monthly thereafter (**Figure 4A**). Interestingly, all 48 female sheep across both groups (25 vaccinated and 23 controls) became pregnant during the study and gave birth without any complications.

Four animals from the pDNA-RVFV-GPC group died on days 146 and 206 post-vaccination (**Table 1**). Serological, neutralization and RT-qPCR analysis at the time of death showed no evidence of RVFV infection in these animals, and they were therefore excluded from subsequent analysis. Unfortunately, no pathological or necropsy investigations were performed, and further investigation could not be conducted. This DNA vaccine platform has previously been used in an animal study without reported mortality (Babuadze et al., 2021), and according to literature, only one RVF DNA-vaccine study reported two deaths in the control group rather than in the vaccinated animals (Chrun et al., 2018). Although vaccination-related causes cannot be entirely excluded, these deaths are more likely attributable to environmental factors. Notably, two of the deaths occurred in late October and two in December, a period in northern Senegal associated with declining pasture quality and increased risk of dietary shifts, digestive disturbances, or exposure to natural toxins (Cooke et al., 2025; Lo et al., 2022). While these environmental factors may have contributed, the exact cause of death remains undetermined in the absence of toxicological or pathological evaluation.

**Table 1:** Description of experimental groups.

Group	Sex			Age (months)			Mortality
	M	F	Total	Min	Max	Median Age	n (day post vaccination)
Control	12	23	35	4	18	8	0 (N.A)
Vaccinated	10	25	35	4	15	8	4 (146, 146, 206, 206)

The sex, age distribution and mortality in vaccinated and control sheep.

The antibody response in the remaining 31vaccinated animals was measured by IgG ELISA, using 100-fold diluted sera against RVFV Gn. Specific IgG were detected 28 days after the first vaccine dose, with an average OD405 of 1.29 ± 0.43 (SD). These levels increased to an average OD405 of 2.00 ± 0.33 (SD) on day 56, 28 days after the second dose. Interestingly, RVFV Gn-specific IgG levels fluctuated over time, suggesting frequent RVFV exposure via mosquito bites (**Figure 4B**).

The neutralizing capacity of antibodies from vaccinated sheep was also evaluated at days 56, 146 and 356. The reciprocal titer dilution corresponding to 90% neutralization increased significantly after the boost, rising from 1 to 40.87 ± 10.15 SEM at day 56 (**Figure 4C**). Since neutralizing capacity is a key indicator of protection against RVFV (Connors et al., 2024), a more detailed analysis was performed, as shown in **Table 2**. Not all animals were able to neutralize more than 90% of infection; at day 56, 45.2% (14/31 sheep) reached this threshold. However, 77.4% (24/31 sheep) were capable of neutralizing over 80%. Interestingly, more than half of the vaccinated sheep retained the ability to neutralize over 50% even after 356 days, suggesting that the vaccine induces a strong and durable neutralizing antibody response. The protective efficacy of pDNA-RVFV-GPC was evaluated using the presence of IgG antibodies against the RVFV nucleoprotein (N)as a surrogate marker of infection. Over the course of a year post-vaccination, only one vaccinated animal developed N-specific antibodies, beginning on day 236. Interestingly, this animal also failed to mount a strong neutralizing antibody response, reaching only 50% neutralization at day 56, which may correlate with reduced protection. In contrast, 5 of the 35 control animals (5/35) showed signs of RVFV infection, as indicated by the production of N-specific IgG. However, the difference in infection rates between the groups did not reach statistical significance (*p* = 0.11), as shown in the graph depicting the percentage of uninfected animals over time. (**Figure 4D**).

**Table 2:** Detailed analysis of sheep neutralizing assay data.

		Day 56	Day 146	Day 356
Number of serum samples		31	30	30
Calculated Reciprocal Titer Mean	ID50	425,7	53,5	45,43
	ID80	93,09	4,063	5,933
	ID90	40,87	2,633	2,633
Number of animals with NeuAb (%)	ID50	31/31 (100%)	20/30 (66,7%)	17/30 (56,7%)
	ID80	24/31 (77,4%)	2/30 (6,7%)	2/30 (6,7%)
	ID90	14/31 (45,2%)	1/30 (3,3%)	1/30 (3,3%)

The number of serum samples analyzed on days 56, 146 and 356 is indicated. The mean reciprocal titers required to achieve 50% (ID50), 80% (ID80) and 90% (ID90) neutralization of RVFV-pseudotyped are reported, along with the number of animals exhibiting neutralizing antibodies (NeuAb) potency.

## Discussion

RVFV mainly circulates among livestock in low- and middle-income countries (LMICs). A veterinary vaccine is, therefore, the most cost-effective strategy to limit RVFV transmission and reduce the risk of spillover events into human populations (Faburay et al., 2017; Fawzy and Helmy, 2019). The two-dose pDNA-RVFV-GPC vaccine candidate developed in this study aligns with key requirements outlined in the WHO’s target product profile for RVFV vaccines (Monath et al., 2020). Although a single-dose vaccine is recommended by the WHO for veterinary use in ruminants, up to two doses may be administered with a short interval between them (Organization, W.H, 2019). The pDNA-RVFV-GPC vaccine candidate demonstrated both safety and efficacy, including in all pregnant animals (25 sheep), with no cases of abortion, stillbirth, or fetal deformities observed, regardless of gestation stage. Additionally, this vaccine is unlikely to affect milk and meat production or pose risks to non-target species. The vaccine-induced immune response can be readily distinguished from that of natural infection. Moreover DNA vaccines are highly stable, including at temperature between 2-8°C, eliminating the need for ultra-cold storage (below -20°C), which is often unavailable in rural areas of low- and middle-income countries (LMICs) (Fanelli et al., 2022).

This vaccine candidate demonstrated a strong capacity to induce robust humoral responses, first in mice and then in sheep under natural field conditions. In vaccinated mice, the IgG endpoint titer reached 1:12,600, representing a notable improvement over previous RVFV DNA vaccine studies, which reported titers below 1:5,000. Although this is lower than titers achieved with live-attenuated vaccines such as MP-12, which can reach up to 1:100,000 (Bhardwaj et al., 2010; Doyle et al., 2022), DNA vaccines offer a superior safety profile. After two doses, this DNA vaccine candidate elicited strong neutralizing antibody responses, with ID90 titers of 1:116 in mice and 1:40 in sheep. According to a previous study in sheep, a PRNT80 titer of 1:40 was sufficient for protection following vaccination with a glycoprotein subunit vaccine (Faburay et al., 2014). Our results showed a titer of 1:93 at 80% neutralization, and fewer animals in the vaccinated group developed RVFV N-specific IgG during the study, only one sheep compared to five in the control group, supporting the conclusion that this vaccine candidate is capable of conferring protective immunity against RVFV under field condition.

Another important feature of this DNA vaccine candidate is the use of a consensus sequence, designed to confer protection against multiple strains of the virus. The sequences used include those from Lineage C (MP-12 and ZH548), as well as sequences from the most prevalent lineage in northern Senegal (Lineage H). Serological assays were performed using recombinant Gn protein from the MP-12 strain, and results under field conditions suggest that this DNA vaccine candidate can protect against the RVFV lineage circulating in northern Senegal. These findings support the potential of the consensus sequence to confer cross-protection against multiple lineages. However, further experiments are needed to confirm the full breadth of strain and lineage coverage provided by this vaccine. For broad adoption, veterinary vaccines against RVFV must be affordable (O’Neill et al., 2024). Especially in LMIC, elevated cost is a barrier to wide adoption of veterinary vaccines against RVFV (Fawzy and Helmy, 2019). Vaccine-grade plasmid DNA can readily be manufactured at low cost (Kisakov et al., 2024). In response to the coronavirus disease 2019 (COVID-19) pandemic, mRNA manufacturing capacity, which includes the production of highly pure plasmid DNA, is developed in LMIC as part of the WHO mRNA technology transfer programme (Gsell et al., 2023). As a result, pDNA-RVFV-GPC could be produced in LMIC, further reducing its cost. The developed pDNA-RVFV-GPC vaccine requires electroporation to increase its immunogenicity. The availability and cost of existing electroporation devices could impede the adoption of our pDNA-RVFV-GPC vaccine (Kisakov et al., 2024). It is worth noting that new, more affordable devices are under-development for the administration of DNA vaccines (Lallow et al., 2021; Xia et al., 2021). Among them, an ultralow-cost handheld electroporator was developed by Xia and colleagues (Xia et al., 2021). Demonstration of the protective efficacy of pDNA-RVFV-GPC administered using a more affordable delivery system is therefore warranted.

In addition to vaccine cost, practical considerations including vaccine hesitancy, efficient vaccine distribution in remote rural areas, availability of trained personnel for vaccine administration, are critical for the success of any vaccination campaign. Collaborations with local and international institutions as well as community engagement would be required to ensure equitable vaccine access in all at-risk areas.

Overall, this study demonstrates the immunogenicity and protective efficacy of a two-dose DNA-based veterinary vaccine against RVFV under real-world conditions. This study warrants future comparisons of the developed DNA vaccines to currently approved RVFV veterinary vaccines (Labeaud, 2010). Larger-scale efficacy trials will be required to assess the long-term impact of RVFV veterinary vaccination on viral prevalence in livestock, humans and mosquitos. In conclusion, this study highlights the potential of the pDNA-RVFV-GPC vaccine to protect immunized livestock in RVFV endemic regions.

## Data Availability

The original contributions presented in the study are included in the article/**Supplementary Material**. Further inquiries can be directed to the corresponding author.

## Supplementary material

Supplementary Figure 1 Representative image of RVFV plaque reduction neutralization assay (PRNT) using PS cell monolayers. Serial two-fold dilutions of sheep serum (10^-^² to 10^-10^) were incubated with 1,000 PFU of RVFV (Smithburn strain) and overlaid on PS cells. Cytopathic effect was visualized by staining with Amido Black. Plaques appear as distinct clear zones against the blue-stained monolayer. CP = Positive control; CN = Negative control.
