# Supplementary material for: A DNA vaccine candidate provides protection against Rift Valley Fever virus in sheep under natural field conditions
Source: Front Cell Infect Microbiol. 2025 Aug 26;15:1628877. doi: 10.3389/fcimb.2025.1628877 (PMC12417402; doi:10.3389/fcimb.2025.1628877)
Supplement: Supplementary Figure 1 — Representative image of RVFV plaque reduction neutralization assay (PRNT) using PS cell monolayers. Serial two-fold dilutions of sheep serum (10-² to 10-10) were incubated with 1,000 PFU of RVFV (Smithburn strain) and overlaid on PS cells. Cytopathic effect was visualized by staining with Amido Black. Plaques appear as distinct clear zones against the blue-stained monolayer. CP = Positive control; CN = Negative control. [file DataSheet1.docx]

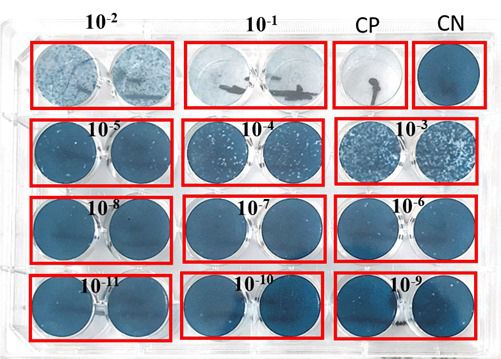


**Supplementary FIgure 1: Representative image of RVFV plaque reduction neutralization assay (PRNT) using PS cell monolayers.**

Serial two-fold dilutions of sheep serum (10⁻² to 10⁻¹⁰) were incubated with 1,000 PFU of RVFV (Smithburn strain) and overlaid on PS cells. Cytopathic effect was visualized by staining with Amido Black. Plaques appear as distinct clear zones against the blue-stained monolayer. CP = Positive control; CN = Negative control.
